# Polysaccharides from Bamboo Shoot (*Leleba oldhami* Nakal) Byproducts Alleviate Antibiotic-Associated Diarrhea in Mice through Their Interactions with Gut Microbiota

**DOI:** 10.3390/foods11172647

**Published:** 2022-08-31

**Authors:** Canhui Chen, Xuefang Guan, Xiaoyan Liu, Weijing Zhuang, Yiqian Xiao, Yafeng Zheng, Qi Wang

**Affiliations:** 1 Institute of Agricultural Engineering, Fujian Academy of Agricultural Sciences, Fuzhou 350003, China; 2 College of Food Science, Fujian Agriculture and Forestry University, Fuzhou 350002, China; 3 Beijing Engineering and Technology Research Center of Food Additives, School of Food and Health, Beijing Technology and Business University, Beijing 100048, China; 4 Fujian Key Laboratory of Agricultural Product (Food) Processing, Fuzhou 350002, China

**Keywords:** polysaccharides, bamboo shoot, gut microbiota, antibiotic-associated diarrhea

## Abstract

A water-soluble polysaccharide BSP was extracted from the basal part of bamboo shoot, a main by-product of bamboo shoot processing. BSP is composed of glucose (72.8%), xylose (19.43%) and a small amount of galactose, arabinose, glucuronic acid and mannose. The effects of BSP on mice with antibiotic-associated diarrhea (AAD) were investigated. The mice fed with BSP exhibited significant higher bodyweight gain, lower pH value and higher concentrations of SCFAs in the feces compared with those fed with saline. BSP administration reduced the inflammatory cells in the small intestine and colon in the AAD mice, and *Firmicutes*/*Bacteroidetes* ratio in the gut was decreased from 0.56 to 0.19. Moreover, BSP administration affected the composition and diversity of the gut microbiota in the AAD mice, particularly on the improvement of beneficial bacteria such as *Bacteroides*, *Lactobacillus* and *Lachnospiraceae_NK4A136_group*. Our results suggest that the polysaccharides from bamboo shoot by-products could be an attractive natural component for gut health and AAD treatment.

## 1. Introduction

Increasing evidence suggests that gut microbiota plays a critical role in human health and disease by modulating the host immune system and metabolism [1,2]. Recent studies discovered that the gut microbiota composition and its balance are closely related to various human diseases, including metabolic disorders such as obesity and diabetes [3], and also mental disorders such as depression and anxiety [4,5]. Short chain fatty acids (SCFAs) are the main metabolites produced by the gut microbiota and captured by the colonic mucosa to provide energy for the host and interacting with membrane receptors and nutrient sensors to regulate various physiological processes [6]. When the abundance and diversity of gut microbiota are negatively affected by diets, living style or antibiotic treatment, the concentrations of SCFAs are significantly decreased and thus fail to perform their functions [7,8].

Antibiotic-associated diarrhea (AAD) is one of the most common clinical complications resulting from antibiotic-damaged gut microbiota s [9]. Antibiotics have been widely used for the treatment of various bacterial infectious diseases. However, the abuse of antibiotics has been proved to cause serious clinical complications [10]. Adjusting or recovering the gut microbiota is an available method for the treatment of AAD [11]. Plant polysaccharides attracted increasing interest due to their ability of escaping digestion in the small intestine and being fermented in the colon, thus enhancing the abundance of beneficial bacteria and inhibiting microbial dysbiosis [12,13]. Recent studies demonstrated that the polysaccharides from *Schisandra chinensis* [14] and *Panax ginseng* [15] exhibited beneficial effects in animals with AAD by increasing SCFA contents and improving the abundance and diversity of gut microbiota. Therefore, screening novel polysaccharides from various plant resources, especially processing by-products, could be a cheap, safe and promising way for the prevention and treatment of AAD and other gut microbiota-related diseases. 

Bamboo, belonging to the Gramineae Bambusoideae, is a valuable evergreen woody grass widely distributed around the world, especially in China and Japan [16,17]. Bamboo shoots are the new edible bamboo culms that just come out of the ground. Owing to their beneficial health properties such as antioxidant [18], hypolipidemic [19] and anti-aging activities [20], bamboo shoots have been widely used as a functional food and traditional Chinese medicine throughout history. *Leleba oldhami* Nakal is one of most economically worthy species of bamboo shoot in southern China and has been developed into various products, such as soft drinks, fermented and canned foods [21]. However, only the tender part on the top of bamboo shoots can be utilized in the food industry, while a huge amount of the basal part of bamboo shoots are discarded, causing resource waste and environmental pollution. Bamboo shoots and their by-products have been proved to be an abundant, cheap and natural source for the production of polysaccharides [22], which have been demonstrated to exhibit prebiotic activity and increasing the production of SCFAs in vitro [23,24]. However, there is still no study on the in vivo effects of bamboo shoot polysaccharide on gut microbiota and the metabolites.

Thus, in this study, a polysaccharide BSP was extracted from the basal part of bamboo shoots. After its chemical composition was characterized, the in vivo effect of BSP on the AAD mice was evaluated by measuring the changes of bodyweight, pH value and SCFAs in the fecal samples and the abundance and diversity of gut microbiota.

## 2. Materials and Methods

### 2.1. Materials and Reagents

The basal parts of bamboo shoots (*Leleba oldhami* Nakal), obtained from Nanping, Fujian province, China, were cut into small pieces, dried in an oven at 55 °C and pulverized into fine powder. The powder was then decolored and defatted with petroleum ether and ethanol and dried for polysaccharide extraction. The monosaccharide standards were obtained from Sigma Company (St. Louis, MO, USA). The short chain fatty acid standards (acetic acid, propionic acid, butyric acid and valeric acid) were purchased from Shanghai Macklin Biochemical (Shanghai, China). Lincomycin hydrochloride was purchased from Shanghai Xinyijinzhu Pharmaceutical Co., Ltd. (Shanghai, China). All the other chemicals used in this study were of analytical grade.

### 2.2. Extraction of the Polysaccharides

The polysaccharides were extracted from pretreated powder according to a previously reported ultrasound-microwave-assisted extraction method with modifications [25]. Briefly, the powder samples were mixed with distilled water with a liquid/solid ratio of 30:1 (*v*/*w*), and then subjected to the ultrasound-microwave-assisted extraction device (XH-300B, Beijing Xianghu, Beijing, China) under the processing conditions of 300 W microwave power, 600 W ultrasonic power and 170 s extraction time. The resulting mixtures were further extracted in a water bath of 95 °C for 2 h and then precipitated with ethanol and deproteinated in Sevag reagent [26]. The polysaccharide named as BSP was obtained after freeze-drying. 

### 2.3. Chemical Component Analysis

The total carbohydrate contents of BSP were measured by the phenol-sulfuric acid colorimetric method. The uronic acid and protein contents were determined by the m-hydroxybiphenyl method and the Coomassie blue protein method, respectively. The analysis of monosaccharide composition of BSP was carried out using the Ion Chromatography (ICS5000, Thermo Fisher Scientific, Waltham, MA, USA). After the BSP sample was hydrolyzed by 2 mol/L trifluoroacetic acid, the hydrolysate was separated on a CarboPac PA20 column using 100 mM/L NaOH-200 mmol/L NaAC-deionized water as mobile phase. The column temperature was 25 °C, and the flow rate was 0.5 mL/min.

### 2.4. Determination of Molecular Weight of BSP

The molecular weight (Mw) of BSP was measured using the Viscotek TDAmax system (Malvern, UK) with a pair of gel-filtration chromatographic columns (300 × 7.8 mm) of A6000M under a flow rate of 0.7 mL/min. The BSP sample was dissolved overnight in 0.1 M NaNO_3_ to a concentration of 2.0 mg/mL, with an injection volume of 100 μL. The detector and column temperature were set as 40 °C. The standard curve was established using polyethyleneglycol (Malvern, UK).

### 2.5. Animal Experimental Design

Male Kunming mice (4 weeks old, 20.0 ± 2.0 g) were purchased from Shanghai Laboratory Animal Center (Shanghai, China) and housed under a controlled temperature of 25 ± 1 °C, a humidity of 50 ± 10% and 12 h/12 h light-dark cycles. The mice were allowed free access to standard diet and water. All animal experiments were performed in strict accordance with EU Directive 2010/63/EU for animal experiments, and approved by the Animal Care Review Committee, Fujian Agriculture and Forest University, China (FAAS18-0029).

After a 7-day acclimation period, 36 mice were randomly distributed into a blank control group (BC, 6 mice) and antibiotic-associated diarrhea group (AAD, 30 mice). In the following 3 days, the mice of the AAD group were administrated with 0.3 mL lincomycin hydrochloride (300 mg/mL) by gastric gavage, twice a day, at 9:00 am and 17:00 pm, while the mice of the BC group accepted an equal volume of physiological saline by gastric gavage. In the following 15 days, the AAD mice were randomly assigned into 5 groups (6 mice/group): the positive control group (PC) and the negative control group (NC) were administrated with inulin (200 mg/kg BW) and the same volume of physiological saline, while low-dosage BSP group (LP), middle-dosage BSP group (MP) and high-dosage BSP group (HP) were administrated with 100, 200 and 400 mg/kg BW of BSP, respectively. Exactly 24 hours after the last gastric gavage, the mice fecal samples were collected and stored at -80 °C. In addition, the mice were sacrificed by cervical dislocation, and the colon and small intestine were collected and cleaned with sterile water and fixed with formalin (10% *v*/*v*) for further histological observation. 

### 2.6. Analysis of Mice Bodyweight and Fecal pH 

During the experiment, the bodyweights of mice were measured every 3 days. The pH values of mice feces were determined with a portable pH meter (METTLER—Toledo, Shanghai, China) every 3 days. 

### 2.7. Analysis of Short Chain Fatty Acids (SCFAs)

The fecal sample (0.5 g) was put into a centrifuge tube in an ice-cold water bath and mixed with 4 mL distilled water by vortex mixer. The tube was cooled in the ice-cold water bath for 20 min and then centrifuged at 5000 rpm for 20 min at 4 °C. The supernatant was collected into another tube, and this process was repeated once. The supernatant was filtered by filter membrane (0.45 μm) and injected in an Agilent 7890A gas chromatography (Palo Alto, CA, USA) with a DB-WAX column (30 m × 0.320 mm × 0.25 μm) for analysis of SCFAs (acetic, propionic, butyric and valeric acids). The injection volume was 0.2 μL, and the initial column temperature was kept at 100 °C for 1 min and then raised to 200 °C at a rate of 8 °C/min. The flow rates of carrier gas (nitrogen), oxidant gas (air), fuel gas (hydrogen) and nitrogen gas were 20, 400, 30, 20 mL/min, respectively. The temperature of the injector and flame ionization detector was maintained at 240 °C.

### 2.8. Histologic Observation of Colon and Small Intestine

The colon and small intestine samples were minced and dehydrated with a series of ethanol solutions (75%, 85%, 90%, 95% and 100% ethanol *v*/*v*, respectively), embedded in paraffin and cut into slices (5 μm thickness). The slices were stained with hematoxylin and eosin (HE) and observed by Olympus BX53 Biological Microscope (Tokyo, Japan).

### 2.9. Total DNA Extraction and Illumina High Throughput Sequencing Analysis

Fast DNA SPIN Extraction Kit (MP Biomedicals, Santa Ana, CA, USA) was applied to extract total bacterial genomic DNA from mice fecal samples. The total DNA concentration and purity were characterized by 1% agarose gel electrophoresis.

The V3-V4 region of the 16S rDNA gene was amplified by PCR using the primer pair with barcode (341F: 5′-CCTACGGGNGGCWGCAG-3′, 806R: 5′-GGACTACHVGGGTATCTAAT-3′). The PCR products were separated and purified by using agarose gel electrophoresis and the AxyPrep DNA Gel Extraction Kit (Axygen Biosciences, Union City, CA, USA), and quantified using QuantiFluor-ST (Promega, Madison, WI, USA), all according to the manufacturers’ instructions. Afterwards, the purified PCR products were equivalent mixed, connected to the junction of sequencing and establishing library of sequencing, then subsequently sequenced by the Illumina HiSeq 2500 System.

### 2.10. Bio-Informatics Analysis

R software packages (v3.2.0) and Quantitative Insights Into Microbial Ecology (QIIME) were carried out to analyze sequence data. Operational taxonomic unit (OTU) was used to represent a sequence with a similarity >97%, compared with the template sequence in the Silva database and obtained the taxonomic information for every OTU. In addition, OTU abundance data were used for the calculation of α and β diversity indices via QIIME [27]. The diversity and richness of samples were analyzed by α diversity indices, such as Shannon index and Chao index; β diversity indices identified structural variations of gut microbial communities of the tested groups [28].

### 2.11. Statistical Analysis

Statistical results were expressed as Mean ± standard deviation (S.D.) and performed in triplicates. Data Processing System (DPS, version 7.05; Zhejiang University, Hangzhou, China) software was used as statistical analyses. Analysis of variance (ANOVA) with Duncan’s range were used to compare the difference between groups, and *p* < 0.05 was considered as significant difference. 

## 3. Results

### 3.1. Composition and Molecular Weight of BSP

**Figure 1:**
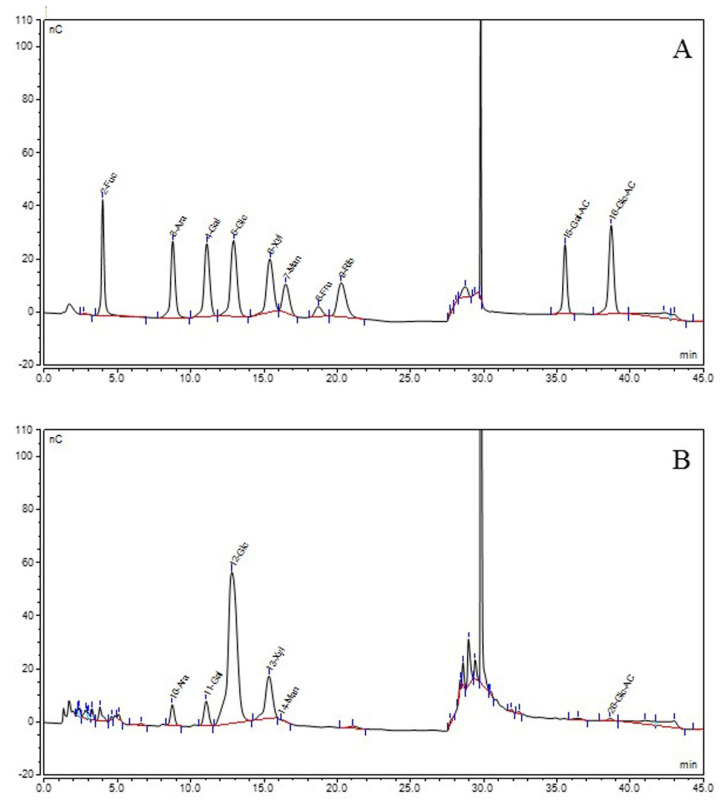
Chromatogram of standard monosaccharide (**A**) and component monosaccharides released from BSPs (**B**).

In this study, the yield of BSP using ultrasound-microwave-assisted extraction method was 10.09% (*w*/*w*), which is significantly higher than the previously reported yield of 6.82% (*w*/*w*) from bamboo shoots using the hot-water method [29]. The contents of total sugar, uronic acid and protein in BSP were 74.87%, 4.05% and 1.89%, respectively. Since the free proteins had been removed by using the Sevag method, the discovered protein was probably bound in the extracted polysaccharides. The results of monosaccharide composition analysis indicated that BSP was composed of 72.8% glucose, 17.9% xylose, 4.4% galactose, 4.4% arabinose, 0.3% glucuronic acid and 0.2% mannose (Figure 1). The average molecular weight of BSP was determined to be 887 kDa.

### 3.2. Effect of BSP on Bodyweight of Mice

**Figure 2:**
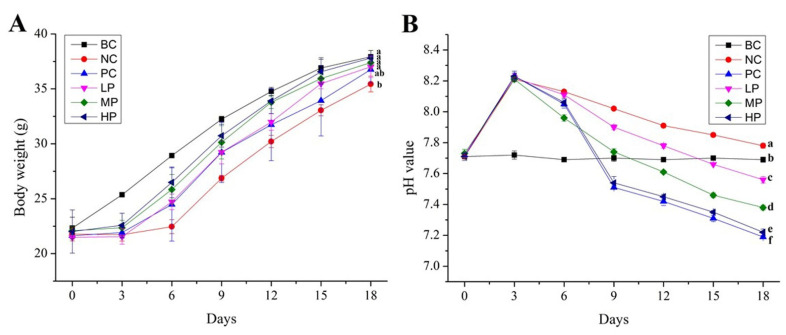
Effects of BSP on bodyweight of mice (**A**) and pH value of mice feces (**B**). BC (blank control) represents healthy mice; NC (negative control) represents AAD mice without treatment; PC (positive control) represents AAD mice administrated with 200 mg/kg BW of inulin; LP, MP and HP represent AAD mice administrated with low-dosage (100 mg/kg BW), middle-dosage (200 mg/kg BW) and high-dosage (400 mg/kg BW) of BSP, respectively. a–f: The results of the day 18 marked without the same letters differed significantly (*p* < 0.05). The values are expressed as means ± S.D.

After lincomycin hydrochloride administration, the AAD mice showed 100% diarrheal symptoms such as lower food intake and frequent loose and watery stools, demonstrating the successful creation of the AAD mice model. The effects of BSP administration on the bodyweight of AAD mice were investigated and shown in Figure 2A. During the 18-d experiment period, the healthy mice in the blank control (BC) group achieved constant bodyweight gain, while the increases of bodyweight of all AAD mice in the model period (0–3 day) were significantly suppressed due to the effect of lincomycin hydrochloride, and the AAD mice receiving physiological saline in the negative control (NC) group increased bodyweight at the lowest rate in the following recovery period (3–18 day). Compared to the mice in NC group, the AAD mice treated with BSP or inulin gradually recovered from diarrhea and achieved a significantly higher rate of bodyweight gain in the recovery period. After 6 d, the average bodyweights of the AAD mice in the high-dosage (HP, 400 mg/kg BW of BSP) and middle-dosage (MP, 200 mg/kg BW of BSP) group were higher than those of the AAD mice in the low-dosage (LP, 100 mg/kg BW of BSP) and the positive control (PC, 200 mg/kg BW of inulin) group mice, while the final bodyweights of experimental mice except for the mice in the NC group showed no significant difference. The results indicated that BSP administration could improve the diarrhea status and increase the bodyweight of AAD mice in a dosage-dependent manner. Interestingly, compared with inulin of 200 mg/kg BW, the same dosage of BSP exhibited more potential on recovering bodyweight of AAD mice.

### 3.3. Effect of BSP on pH Value and SCFAs of Mice Feces 

The pH value of feces can indirectly respond to the pH value of the intestinal environment, which is strongly related with intestinal health. Figure 2B shows the effect of BSP on the pH value of mice feces. Compared with BC group mice, the pH values of AAD mice feces significantly increased in the model period, indicating an unhealthy alkaline intestinal environment suitable for the growth of spoilage bacteria. With the treatment of BSP or inulin, the pH values of AAD mice feces significantly decreased. At the end of the experiment, all treated groups were significantly lower than those of NC and BC groups. Moreover, BSP administration exhibited significant effects on decreasing the pH value of AAD mice feces in a dose-dependent manner, while 400 mg/kg BW of BSP administration had a similar effect as 200 mg/kg BW of inulin administration.

The SCFAs were generated from anaerobic intestinal beneficial bacteria by fermentation of dietary fibers and mainly contained acetic acid, propionic acid, butyric acid and valeric acid. In addition, SCFAs can improve inflammatory reaction and energetic metabolism for host and indirectly react to the activity of gut bacteria. Thus SCFAs are an important index for the state of the intestinal environment [8]. Compared with the mice in BC and NC groups, in mice treated with fermentable inulin or BSP, concentrations of acetic acid, propionic acid, butyric acid and valeric acid were significantly increased (*p* < 0.05, Table 1), which was consistent with the decreased pH value in the mice feces (Figure 2B). Moreover, compared with the mice treated with inulin of 200 mg/kg BW, in the AAD mice treated with high dosage of BSP (400 mg/kg BW), significant higher concentrations of butyric acid and valeric acid were noticed (*p* < 0.05), while no significant differences were found for the concentrations of acetic acid and propionic acid. Among the SCFAs, butyric acid is considered the most important due to its important role in maintaining intestinal function and modulating immune and inflammatory responses in animal models [30].

### 3.4. Histologic Observation of Colon and Small Intestine

**Figure 3:**
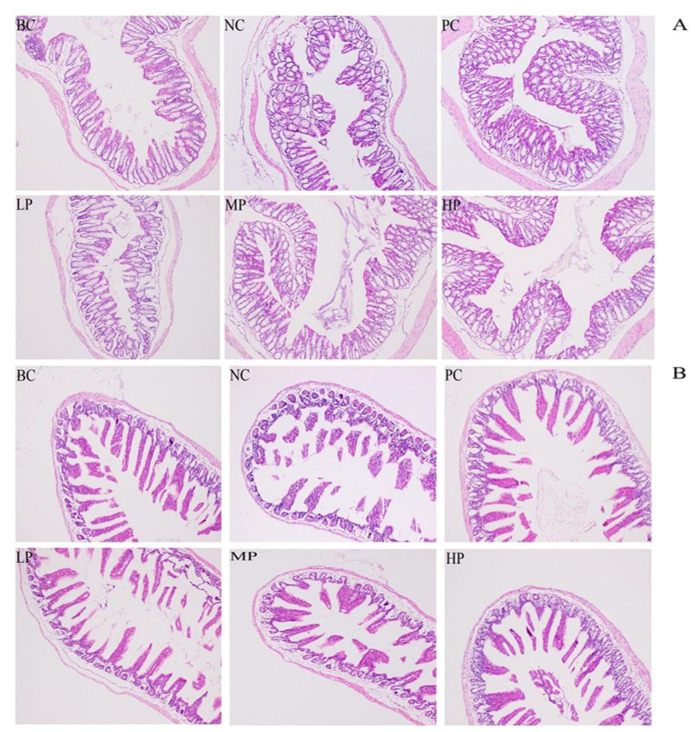
Histopathological observation of the colon (**A**) and small intestine (**B**) (100×). BC (blank control) represents healthy mice; NC (negative control) represents AAD mice without treatment; PC (positive control) represents AAD mice administrated with 200 mg/kg BW of inulin; LP, MP and HP represent AAD mice administrated with low-dosage (100 mg/kg BW), middle-dosage (200 mg/kg BW) and high-dosage (400 mg/kg BW) of BSP, respectively.

Figure 3 shows the histological changes in the colon and small intestine for each group. Normal histological features of the colon and small intestine were observed in the BC group. However, the colonic and small intestinal villous stroma and submucosa of the AAD mice in the NC group presented oedema and abundant inflammatory cell infiltration after antibiotic treatment. Moreover, compared with the BC group, the small intestinal villi were relatively sparse and short, and the intestinal wall was thinner. The morphological damage and inflammatory infiltration could be one of the complex mechanisms by which antibiotics lead to AAD. Compared with the NC group, the treatment of BSP could significantly improve the colonic and small intestinal pathological features with thicker intestinal wall and fewer inflammatory cells. Based on the histologic observation, the HP group showed the most effective recovery function among the BSP-treated groups and showed no significant difference to those of the PC group.

### 3.5. Overall Structural Characteristics of the Mice Microbiome 

**Figure 4:**
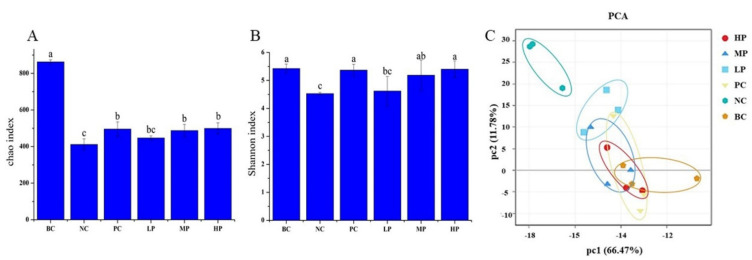
The analysis of abundance and diversity of the gut microbiota: (**A**) Chao index; (**B**) Shannon index; (**C**) principal component analysis (PCA) score plot. BC (blank control) represents healthy mice; NC (negative control) represents AAD mice without treatment; PC (positive control) represents AAD mice administrated with 200 mg/kg BW of inulin; LP, MP and HP represent AAD mice administrated with low-dosage (100 mg/kg BW), middle-dosage (200 mg/kg BW) and high-dosage (400 mg/kg BW) of BSP, respectively. a–c: Graph bars marked without the same letters differed significantly (*p* < 0.05). The values are expressed as means ± S.D.

To verify the influence of BSP on the microbiota in the AAD mice, the HiSeq2500 System was used to sequence the V3-V4 regions of the 16S rDNA gene from mice fecal. A total 3,773,651 of effective tags were obtained from 18 mice fecal samples. The Chao index (Figure 3A) and the Shannon index (Figure 4B) were used to demonstrate the microbial abundance and diversity, respectively [14]. The results indicated that after the treatment of lincomycin hydrochloride, the microbial abundance and diversity in the mice were both dramatically decreased. BSP or inulin could be fermented and utilized by the gut microbiota in the AAD mice, therefore contributing to the recovery of the microbial abundance and diversity. In the mice in the PC, MP and HP groups, the microbial diversities were recovered to the similar level of that of the mice in the BC group. Inulin or BSP administration exhibited significant effects (*p* < 0.05) on the recovery of the microbial abundance in the AAD mice; however, none of them could fully recover the microbial abundance due to the short recovery period (15 d). 

Figure 4C shows the principal component analysis (PCA) score plot, which was used to determine the similarity of microbial communities among the experimental groups [31]. The mice in the BC group had undamaged and healthy gut microbiota; therefore the longer distance to the points of the BC group could be considered as less recovery from damaged gut microbiota in the mice. According to Figure 4C, the points of the NC group were farthest from those of the BC group, suggesting that the mice’s bacterial community slowly recovered after being seriously damaged by antibiotics. Compared with the NC group, the points of the groups receiving BSP or inulin treatment were closer to those of the BC group, indicating the significant recovery function on the gut microbiota in the AAD mice by consuming these fermentable polysaccharides. It was notable that the points of the HP group were the closest to those of BC group. 

### 3.6. Composition Analysis of the Gut Microbiota

**Figure 5:**
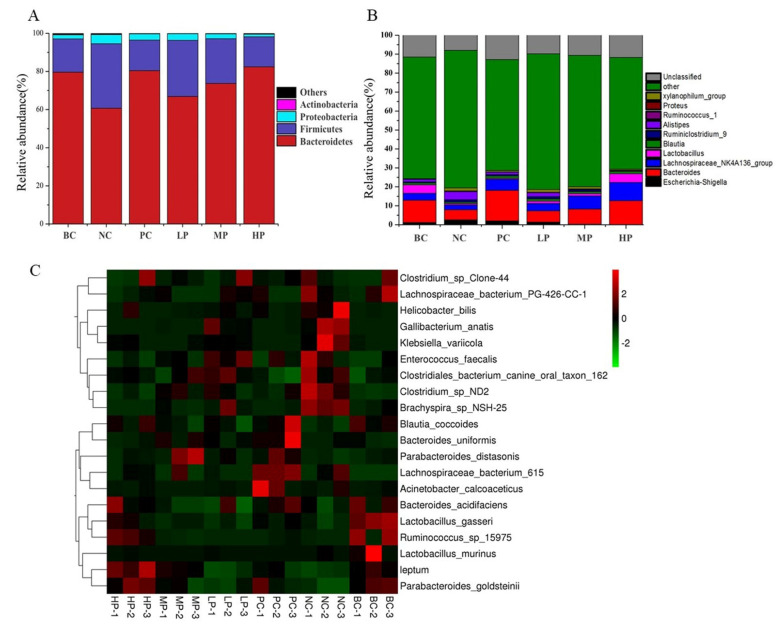
Composition of the gut microbiota represented by the stacking histograms at phylum level (**A**) and genus level (**B**), as well as a heatmap at species level (**C**). BC (blank control) represents healthy mice; NC (negative control) represents AAD mice without treatment; PC (positive control) represents AAD mice administrated with 200 mg/kg BW of inulin; LP, MP and HP represent AAD mice administrated with low-dosage (100 mg/kg BW), middle-dosage (200 mg/kg BW) and high-dosage (400 mg/kg BW) of BSP, respectively.

Based on the analysis results of 16S rDNA gene sequences, the relative abundance of bacterial phylum in different groups is presented as a stacking histogram in Figure 5A. All the experiment groups mainly constituted of *Firmicutes*, *Bacteroidetes* and *Proteobacteria* at the phylum level. Compared with the healthy mice in the BC group, the relative abundance of *Bacteroidetes* in the NC group sharply decreased from 79.61% to 60.72%, while a dramatic two-fold increase was observed in both *Firmicutes* and *Proteobacteria*, confirming the disruptive changes in the gut microbiota (dysbiosis) caused by antibiotic treatment. Both inulin and BSP administration improved gut dysbiosis after 15 days of recovery period, and BSP exhibited its recovery effect in a dose-dependent manner. Both inulin of 200 mg/kg BW and BSP of 400 mg/kg BW could recover the relative abundance of Bacteroidetes and Firmicutes to their respective normal levels. The corresponding *Firmicutes*/*Bacteroidetes* ratio decreased from 0.56 (NC group) to 0.20 (PC group) and 0.19 (HP group), indicating that *Firmicutes* was effectively inhibited by inulin and BSP [32]. However, the relative abundance of *Proteobacteria* remained elevated in the mice treated with inulin, while BSP treatment proved to be more effective on inhibiting *Proteobacteria*. 

The effects of BSP administration on the gut microbiota of the AAD mice were further investigated at the genus level (Figure 5B). Comparing the main differences of the gut microbiota at the genus level between the BC and NC groups, it was found that the relative abundance of genera of *Bacteroides*, *Lactobacillus* and *Lachnospiraceae_NK4A136_group* were significantly decreased, whereas the relative abundance of genera of *Escherichia-Shigella*, *Ruminiclostridium_9*, *Ruminococcus_1*, *xylanophilum_group*, *Alistipes* were increased. The 15 days of BSP or inulin treatment improved gut dysbiosis at different levels by reversing the altered abundance of genera to relatively normal levels. Interestingly, compared with inulin, BSP was observed have more potential on promoting *Lactobacillus* and *Lachnospiraceae_NK4A136_group*, but less efficient on *Bacteroides*. In summary, BSP treatment can regulate the composition and diversity of the gut microbiota in the AAD mice, and thus alleviate the symptoms of diarrhea.

Results of the heat map analysis showed differences in gut microbial richness at the species level (Figure 5C). After the administration of BSP (400 mg/kg BW) for 15 days, the gut microbiota of the AAD mice in HP groups was rich in *Lactobacillus_gasseri*, *leptum*, Bacteroides_acidifaciens, Parabacteroides_distasonis, Lactobacillus_murinus and Rumi*nococcus_sp_15975*, whereas that of the AAD mice in NC groups administrated with only saline was rich in *Clostridium_sp_ND2*, *Helicobacter_bilis*, *Enterococcus_faecalis*, *Brachyspira_sp_NSH-25*, *Gallibacterium_anatis*, *Klebsiella_variicola*, *Clostridiales_bacterium_canine_oral_taxon_162*.

## 4. Discussion

It is well recognized that the gut microbiota composition and its balance are controlled and influenced not only by host genetics, but also by various environmental factors [33]. Polysaccharides have been considered as main components in most herbal plants, and reported to ameliorate different diseases by modulating gut microbiota [6,15]. Bamboo shoot, a vegetable also widely used as a traditional Chinese herb, has attracted increasing attention recently. Bamboo shoot polysaccharides were found to have the possibility to modulate gut microbiota in vitro [13].

Dysbiosis of gut microbiota is a main phenomenon for AAD, which can be easily induced by antibiotic treatment. To investigate the effects of BSP on the dysbiosis, we established a model of AAD mice induced by lincomycin hydrochloride. The results of our study demonstrated that AAD mice showed typical symptoms, including increased number of defecations and diarrhea, lower food intake, decreased actions and listless state. The inflammatory cells were found in the small intestine and colon in the AAD mice. The increased pH values of the feces were observed and correlated with the significantly decreased concentrations of SCFAs. In these AAD mice, alterations in the composition of the gut microbiota at phylum level were also noticed with increasing the relative abundance of *Firmicutes* and *Proteobacteria* and decreasing the relative abundance of *Bacteroidetes*. Meanwhile, the abundance and diversity of the gut microbiota in the AAD mice were dramatically decreased. All this evidence demonstrated the damaged gut structure, imbalanced microbiota and its metabolites in the AAD mice.

BSP administration ameliorated AAD in the mice in a dose-dependent manner. The mice treated with BSP recovered bodyweight, decreased pH values and increased the contents of SCFAs in the feces. The inflammation in the small intestine and colon also gradually recovered. Moreover, BSP recovered the gut microbial diversity and abundance. The comparison of the gut microbiota in mice from the BSP-treated groups and the NC group (without treatment) offered further insights on how BSP alters the microbial environment. BSP administration decreased the corresponding *Firmicutes*/*Bacteroidetes* ratio from 0.56 (NC group) to 0.19 (HP group), suggesting that BSP could efficiently enrich the abundance of *Bacteroidetes* and decrease that of *Firmicutes*. It was also notable that BSP could significantly inhibit *Proteobacteria*, which includes a wide variety of pathogens. The results demonstrated the role of BSP in the re-establishment of a normal microbial environment at the phylum level. Previously reported polysaccharides isolated from *Laminaria japonica* (LP) [3] also proved to be able to decrease the abundance of *Proteobacteria*, while they failed to decrease the corresponding *Firmicutes*/*Bacteroidetes* ratio. Therefore, BSP could be a better polysaccharide compare to those extracted from other plant resources.

At the genus level, BSP increased the abundance of *Bacteroides*, *Lactobacillus* and Lach*nospiraceae_NK4A136_group* but decreased the abundance of *Escherichia-Shigella*, *Ruminiclostridium_9*, *Ruminococcus_1* and *Proteus* compared to mice in the NC group. Among those enriched bacteria, *Bacteroides*, belonging the family *Bacteroidaceae*, has been related to the production of both acetic and propionic acids in the colon [7]; *Lactobacillus* has been well recognized as a beneficial bacteria for the treatment of diarrhea due to its effects on the homeostasis of the microbial environment [15]; *Lachnospiraceae_NK4A136_group* belongs to the order *Clostridiales*, which can ferment diverse plant polysaccharides to SCFAs and alcohols [34]. *Escherichia-Shigella*, genetically closely related to *E. coli.*, is a leading bacterial cause of diarrhea [35], which was significantly inhibited by BSP. Our results revealed that BSP affected the composition and diversity of the gut microbiota in the AAD mice, particularly on the improvement of beneficial bacteria. In another similar study [14], *Schisandra chinensis* polysaccharides were found to affect rats with AAD through increasing the abundance of other beneficial bacteria including *Blautia*, *Intestinibacter* and *Lachnospiraceae-UCG-008*. Therefore, it could be worthy of further investigation on using multiple polysaccharides for the AAD treatment.

SCFAs, the main metabolites of gut microbiota, are produced after undigested dietary components such as plant polysaccharides fermented in the colon. SCFAs have diverse physiological roles in maintaining human health relating to immune-regulation, anti-inflammatory activity and inhibition of the overgrowth of potentially pathogenic bacteria [36]. A significant decrease of SCFAs was noticed in the AAD mice, which could be due to the decrease of SCFA-producing bacteria. It was found that BSP significantly improved the concentrations of acetic acid, propionic acid, butyric acid and valeric acid in the mice feces compared to the NC group. The result could be well explained by the fact that BSP increased the abundance of beneficial bacteria involved in SCFA production.

## 5. Conclusions

In conclusion, our study suggests that basal parts of the bamboo shoot can be used as a valuable resource for the production of BSP, which was demonstrated to be able to positively affect the gut microbiome structure in the AAD mice, and thus ameliorated the syndrome. Specifically, BSP enriched the abundance of beneficial bacteria and inhibited harmful bacteria. Although the mechanism of its prebiotic effect and its relationship with the polysaccharide structure need to be further investigated in our future studies, the present results provide evidence that BSP could be used as a functional ingredient for the prevention and treatment of AAD and other gut dysbiosis related metabolic diseases, such as obesity, diabetes and hyperlipidemia.

## Figures and Tables

**Table 1:** Effect of BSP on SCFAs of mice feces (μmol/g).

Groups	Acetic Acid	Propionic Acid	Butyric Acid	Valeric Acid
BC	94.78 ± 3.06 ^c^	11.69 ± 0.47 ^c^	8.70 ± 0.33 ^e^	0.98 ± 0.05 ^c^
NC	84.23 ± 1.92 ^d^	7.78 ± 0.37 ^d^	6.29 ± 0.10 ^f^	0.75 ± 0.07 ^d^
PC	109.67 ± 3.17 ^a^	14.12 ± 0.34 ^a^	11.35 ± 0.38 ^b^	1.13 ± 0.03 ^b^
LP	96.57 ± 3.57 ^c^	11.31 ± 0.30 ^c^	9.09 ± 0.23 ^d^	0.98 ± 0.03 ^c^
MP	101.90 ± 2.24 ^b^	12.32 ± 0.14 ^b^	9.60 ± 0.12 ^c^	1.14 ± 0.04 ^b^
HP	107.27 ± 1.40 ^a^	13.60 ± 0.22 ^a^	12.50 ± 0.17 ^a^	1.63 ± 0.04 ^a^

BC (blank control) represents healthy mice; NC (negative control) represents AAD mice without treatment; PC (positive control) represents AAD mice administrated with 200 mg/kg BW of inulin; LP, MP and HP represent AAD mice administrated with low-dosage (100 mg/kg BW), middle-dosage (200 mg/kg BW) and high-dosage (400 mg/kg BW) of BSP, respectively. ^a–f^: Data within the same column without the same superscript differed significantly (*p* < 0.05). The values are expressed as means ± S.D.

## Data Availability

Data is contained within the article.
